# The expression of matrix metalloproteinase 2, 9 and 11 in Ethiopian breast cancer patients

**DOI:** 10.1186/s13104-023-06518-5

**Published:** 2023-10-05

**Authors:** Esmael Besufikad Belachew, Adey Feleke Desta, Dinikisira Bekele Deneke, Tewodros Yalew Gebremariam, Dessalegn Abeje Tefera, Fikadu Alemu Atire, Dawit Hailu Alemayehu, Tamirayehu Seyoum, Marcus Bauer, Selfu Girma, Dareskedar Tsehay Sewasew, Eva J. Kantelhardt, Tesfaye Sisay Tessema, Rawleigh Howe

**Affiliations:** 1 Biology Department, College of Natural and Computational Sciences, Mizan Tepi University, Mizan, Ethiopia; 2 Department of Microbial, Cellular and Molecular Biology, College of Natural and Computational Sciences, Addis Ababa University, Addis Ababa, Ethiopia; 3 Armauer Hansen Research Institute, Addis Ababa, Ethiopia; 4 Department of Pathology, School of Medicine, College of Health Science, Tikur Anbessa Specialized Hospital, Addis Ababa University, Addis Ababa, Ethiopia; 5 Global Health Working Group, Martin Luther University Halle-Wittenberg, Halle (Saale), Germany; 6 Institute of Pathology, Martin Luther University Halle-Wittenberg, Halle (Saale), Germany; 7 Department of Gynecology, Martin Luther University Halle-Wittenberg, Halle (Saale), Germany; 8 Institute of Medical Epidemiology, Biostatistics, and Informatics, Martin Luther University Halle-Wittenberg, Halle (Saale), Germany; 9 Institute of Biotechnology, Addis Ababa University, Addis Ababa, Ethiopia

**Keywords:** BC, Benign breast tumor, Ethiopia, Matrix metalloproteinases, mRNA expression

## Abstract

**Introduction:**

Matrix metalloproteinases (MMPs) play a pathophysiological role in cancer initiation and progression. Numerous studies have examined an association between MMP-2, MMP-9, and MMP-11 expression and clinicopathological characteristics of breast cancer (BC); however, no research has been done on the MMP expression levels in BC cases from Ethiopia.

**Materials and methods:**

A total of 58 formalin-fixed paraffin-embedded breast tissue samples encompassing 16 benign breast tumors and 42 BC were collected. The RNA was extracted and quantitative reverse-transcription PCR was performed. GraphPad Prism version 8.0.0 was used for statistical analysis.

**Results:**

The MMP-11 expression levels were significantly higher in breast cancer cases than in benign breast tumors (P = 0.012). Additionally, BC cases with positive lymph nodes and ER-positive receptors had higher MMP-11, MMP-9, and MMP-2 expression than cases with negative lymph nodes and ER-negative, respectively. The MMP-11 and MMP-9 expressions were higher in grade III and luminal A-like tumors than in grade I-II and other subtypes, respectively.

**Conclusion:**

The MMP-11 expression was higher in BC than in benign breast tumors. Additionally, MMP-11, MMP-9, and MMP-2 were higher in BC with positive lymph nodes and estrogen receptors. Our findings suggest an important impact of MMPs in BC pathophysiology, particularly MMP-11.

**Supplementary Information:**

The online version contains supplementary material available at 10.1186/s13104-023-06518-5.

## Introduction

Matrix metalloproteinases (MMPs) are a family of zinc-dependent extracellular matrix remodeling endopeptidases, which play essential roles in physiological processes such as organogenesis, cell repair, remodeling of tissues, apoptosis, and motility [1]. The MMPs are also involved in pathological processes like cancer development, tumor neovascularization, angiogenesis, invasion, and metastasis [2, 3]. The expression and activity of MMPs are increased in advanced tumor stages and metastasized disease [2]. Currently, at least 26 members of this family are known to exist and are divided into four main groups: interstitial collagenases, gelatinases, stromelysins, and membrane-type MMPs [4]. Gelatinase MMPs such as matrix metalloproteinase − 9 (MMP-9) and matrix metalloproteinase − 2 (MMP-2) overexpression are associated with oral cancer, colorectal tumor, bladder carcinoma, retinoblastoma, pancreatic cancer, and ovarian cancer [5–10].

Several studies have investigated the association between clinicopathological features of breast cancer with MMP-2, MMP-9, and matrix metallopeptidases − 11 (MMP-11) expression. There was an inverse correlation between the expression of MMP-2 and MMP-9 in breast cancer [2, 11–14]. There is also a positive correlation between the expression of MMP-2, MMP-9, and MMP-11 and breast cancer prognosis [15–18]. In addition, an earlier study by Chenard and colleagues revealed that MMP-11 levels showed no correlation with breast tumor size, axillary-node status, and tumor grade [19]. Despite these inconsistent results, there is no study conducted on the expression levels of MMPs in breast cancer cases from Ethiopia. This study aims to explore the association between MMP-2, MMP-9, and MMP-11 expression with clinicopathologic features among breast cancer patients in Ethiopia.

## Materials and methods

### Study participants

A total of 58 formalin-fixed paraffin-embedded (FFPE) tissue blocks were collected. 42 were from BC cases from referral hospitals in multiple peripheral regions of Ethiopia (24 from Ayder Referral Hospital (Mekelle City, Tigray region), 8 from Hiwot Fana Specialized University Hospital (Harer City, Hareri Region), 4 from ALERT Specialized Hospital (Addis Ababa city), 3 from Jimma University Specialized Hospital (Jimma city, Oromia region), and 3 from Hawassa University Specialized Referral Hospital (Hawassa city, SNNP region). 16 cases with benign breast tumors were collected from ALERT Specialized Hospital.

### Data collection

The demographic and histopathological data were collected from pathology results in each hospital using a data collection form.

### RNA extraction

**Fig. 1:**
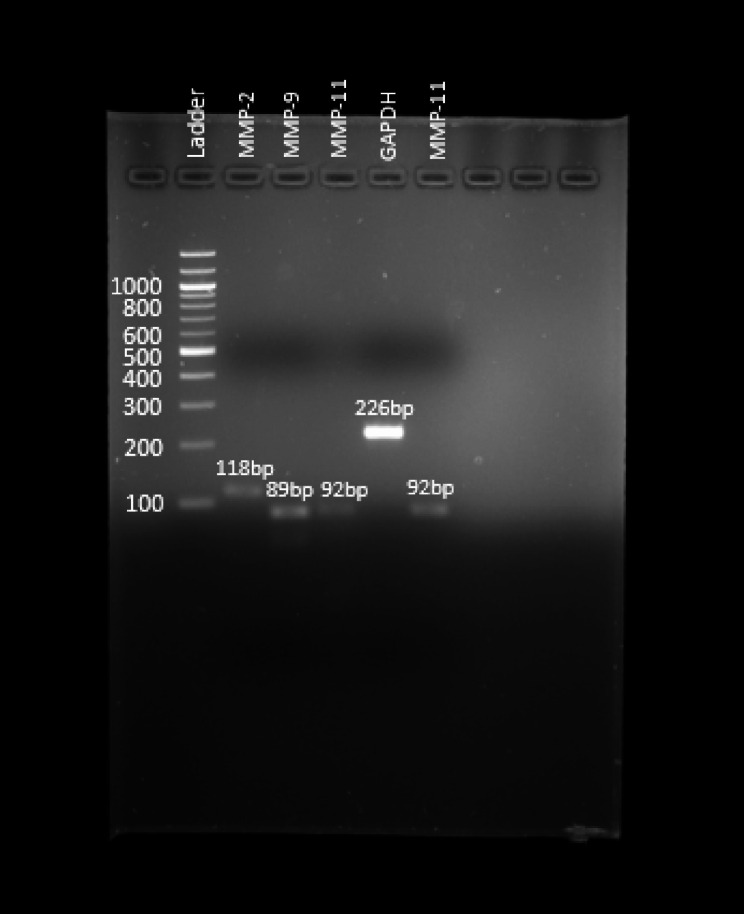
Representative PCR amplification result for MMP-2, MMP-9, MMP-11, and GAPDH

The RNA was extracted from stored FFPE breast tissue specimens using the RNeasy® FFPE Kit (QIAGEN, Hilden, Germany) (Cat No 73,504) following the manufacturer’s protocol. Ten tissue sections of 2 μm thickness per sample were used for RNA extraction. The quality of extracted RNA was checked using a Nanodrop 2000 spectrophotometer. To confirm the presence of the desired PCR product, a standard PCR was performed (Fig. 1). All extracted RNA samples were then stored at -80◦C until the RT-PCR test was performed.

### Quantitative one-step RT-PCR

Specific primers and probes sequence for MMP2, MMP9, and MMP11 were taken from the previous literature [20] and their appropriateness was checked using the Primer-Blast tool in NCBI (Table 1). The PCR reactions were carried out on the CFX96 Deep well Real-time PCR instrument (Bio RAD, Singapore). All quantitative reverse-transcription PCRs were performed in duplicate using the SuperScript™ III Platinum™ One-Step qRT-PCR Kit (Invitrogen/Life Technologies Corporation, Carlsbad, CA 92,008 USA) according to the manufacturer’s instructions. The GAPDH gene was used as an endogenous control. To determine the relative RNA levels of expression within the samples, standard curves for the PCR reactions were performed.

**Table 1:** TaqMan primers and probes sequence for the human MMPs and GAPDH

Gene	Gene bank accession no.		The sequence of primers and FAM-BHQ1 probes	Amplicon size (bp)
MMP-2	NM004530	Forward primer (5’-3’)	TGGCGATGGATACCCCTTT	118
		Reverse primer (5’-3’)	TTCTCCCAAGGTCCATAGCTCAT	
		Probe (5’-3’)	FAMCTCCTGGCTCATGCCTTCGCCCBHQ1	
MMP-9	NM004994	Forward primer (5’-3’)	CCTGGGCAGATTCCAAACCT	89
		Reverse primer (5’-3’)	GCAAGTCTTCCGAGTAGTTTTGGAT	
		Probe (5’-3’)	FAMCTCAAGTGGCACCACCACAACATCACCBHQ1	
MMP-11	NM005940	Forward primer (5’-3’)	CCGCCAGATGCCTGTGA	92
		Reverse primer (5’-3’)	CGGAGGCGCCACACAA	
		Probe (5’-3’)	FAMCCTCCTTTGACGCGGTCTCCACCBHQ1	
GAPDH	NM001357943	Forward primer (5’-3’)	GAAGGTGAAGGTCGGAGTC	226
		Reverse primer (5’-3’)	GAAGATGGTGATGGGATTTC	
		Probe (5’-3’)	FAMCAAGCTTCCCGTTCTCAGCCBHQ1	

### Statistical analysis

Statistical analysis was then performed through GraphPad Prism version 8.0.0 for Windows (GraphPad Software, San Diego, California USA, www.graphpad.com). The assumption of normality was evaluated using the Shapiro normality test. Based on the skewed distribution of the dataset, a non-parametric t-test followed by a Mann-Whitney test was used for the comparison of different groups, and a p-value < 0.05 was considered statistically significant.

## Results

### Socio-demographic and clinical characteristics

A total of 58 study participants were involved in this study, of which 42 (72.4%) and 16 (27.6%) had BC and benign breast tumors, respectively. The mean age at diagnosis was 36.6 (SD ± 13.5) years (Table 2). Grade III BC accounted for 42.9% and the size of T3-T4 accounted for 45.2%. Lymph node positivity was seen in 66.6% of BC cases. The most common histomorphological type was invasive ductal carcinoma (85.7%). Estrogen receptor (ER) and progesterone receptor (PR) positivity was 59.5% and 50.0%, respectively. Human epidermal growth factor receptor-2 (HER2) positivity was 19.0%. The most common immunohistochemistry-defined subtype was the luminal subtype (luminal A and B) which accounted for 47.6% (Table 3).

**Table 2:** Demographic characteristics of study participants with benign breast tumor and BC.

Variables		Frequency	Percent
Age group	15–29	16	28.6
	30–44	26	46.4
	45–59	9	16.1
	≥ 60	5	8.9
	Total	56	100.0
	Missing	2	
	Mean ± Sd (Minimum, Maximum) = 36.6 ± 13.5(15,70)		

**Table 3:** Clinical characteristics of study participants with BC. Differences of features among cases assessed by the Mann-Whitney test

Variables		Frequency (%)	MMP-2	MMP-9	MMP-11
			P-value		
Grade	I-II	24(57.1)	0.8112	0.4423	0.4689
	III	18(42.9)			
	Total	42(100.0)			
Tumor Size	T1-T2	14(33.3)	0.4828	0.5773	0.5708
	T3_T4	19(45.2)			
	Not assessed	9(21.5)			
	Total	42(100.0)			
Lymph node	Positive	28(66.6)	0.5421	0.5656	0.1096
	Negative	9(21.5)			
	Not assessed	5(11.9)			
	Total	42(100.0)			
Histomorphological type	Ductal carcinoma	36(85.7)	0.6611	0.1112	0.0221
	Others	6(14.3)			
	Total	42(100.0)			
ER	Positive	25(59.5)	0.4164	0.1528	0.0514
	Negative	17(40.5)			
	Total	42(100.0)			
PR	Positive	21(50.0)	0.8813	0.7088	0.1123
	Negative	21(50.0)			
	Total	42(100.0)			
HER2	Positive	8(19.0)	0.5913	0.7935	0.6910
	Negative	26(62.0)			
	Equivocal	8(19.0)			
	Total	42(100.0)			
HER2 Score	IHC0	17(40.5)	0.2523	0.3413	0.2499
	IHC 1 + negative	9(21.5)			
	IHC 2 + equivocal	8(19.0)			
	IHC 3 + positive	8(19.0)			
	Total	42(100.0)			
Ki-67	< 20%	20(47.6)	0.1162	0.9505	0.6494
	≥ 20%	22(52.4)			
	Total	42(100.0)			
IHC defined BC subtypes	Luminal A	9(21.5)	0.0706	0.7768	0.6292
	Luminal B	11(26.1)			
	HER2	5(11.9)			
	Triple-negative BC	9(21.5)			
	Not determine	8(19.0)			
	Total	42(100.0)			

### Relative mRNA expressions of MMPs in BC and benign breast tumor cases

**Fig. 2:**
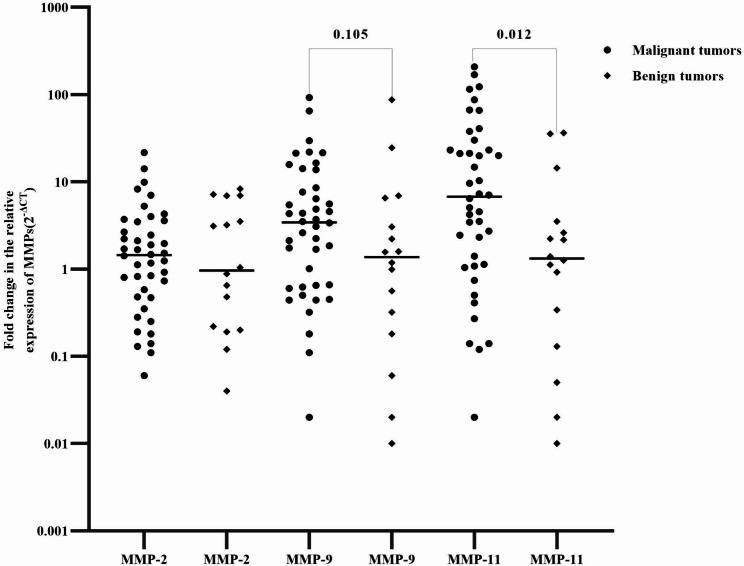
Expression of MMP-2, MMP-9, and MMP-11 in BC and benign breast tumor cases. Fold change in the relative levels of MMP-11 was log-transformed with median values indicated for each group by the horizontal lines

The mRNA expression of MMP-11 was 5.1 times higher in BC than in benign breast tumors cases and the difference was statistically significant (P = 0.012). Higher mRNA expression of MMP-9 was also seen in BC (P = 0.105 (Fig. 2).

### Relative expression of MMP-11 mRNA on BC and benign breast tumors grouped by Ki-67 expression, grade, and lymph node status

**Fig. 3:**
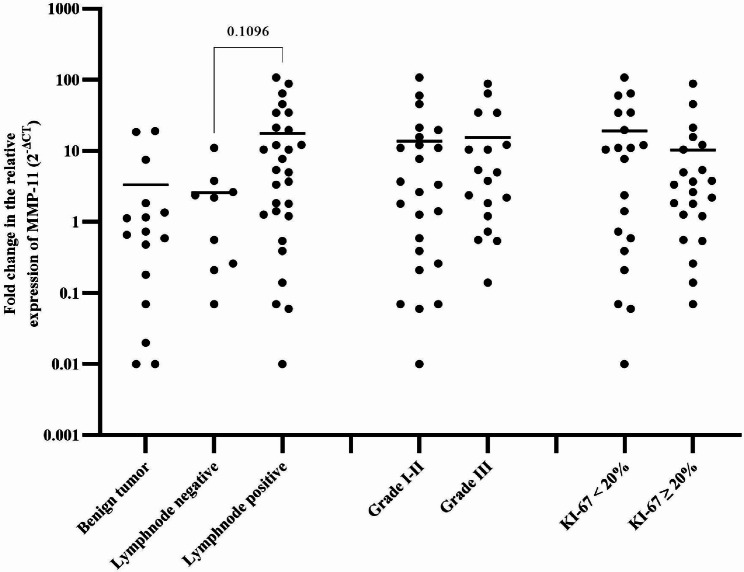
Expression of MMP-11 in cases of benign breast tumor and BC categorized by Ki-67 + cell percentage, grade, and lymph node status. Log transformed values with median are denoted by horizontal lines

The expression of MMP-11 was 2.4 times higher in BC cases with lymph node positivity than in cases with negative lymph nodes (P = 0.1096). The MMP-11 expression was no statistically significant difference compared with grade I or II BC cases (Fig. 3).

### MMP-11 relative mRNA expressions in groups of ER, PR, HER2 status, and subtypes in BC and benign breast tumors

**Fig. 4:**
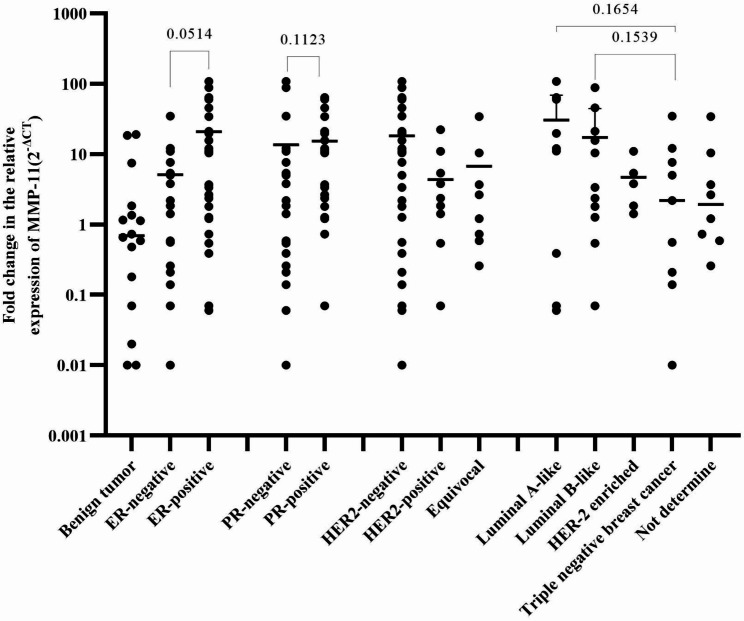
Expression of MMP-11 in cases of benign breast tumors and BC categorized by ER, PR, HER2 status, and IHC-defined BC subtypes. Log transformed values with median are denoted by horizontal lines

The expression of MMP-11 was 5.7 times higher in ER-positive than ER-negative BC cases (P = 0.0514). The MMP-11 expression was 2.4 times higher in HER2-negative BC cases than in HER2-positive cases. Luminal A-like BC subtypes had higher MMP-11 expression than benign breast tumors and other subtypes of BC (Fig. 4).

### MMP-2 relative mRNA expressions of BC and benign breast tumors grouped with Ki-67, grade, lymph node, ER, PR, HER2 status, and subtypes

The BC cases with lymph node-positive had MMP-2 expression levels that were 1.6 times higher than those with lymph node-negative BC. The MMP-2 expression was 2 times higher in KI-67 < 20 cases than in Ki-67 ≥ 20% (see Additional file 1). The MMP-2 expression was 1.3 times higher in HER2-negative BC patients compared to HER2-positive BC cases, but the difference was not statistically significant (see Additional file 2).

### MMP-9 relative mRNA expressions of BC and benign breast tumors grouped with Ki-67, grade, lymph node, ER, PR, HER2 status, and subtypes

The MMP-9 expression was higher in grade III BC cases than in Grade I-II BC cases, with a 1.9 times higher difference (see Additional file 3). The ER-positive BC cases had MMP-9 expression that was 2 times higher than ER-negative BC cases. MMP-9 expression was higher in luminal A-like BC subtypes compared to benign breast tumors and other subtypes (see Additional file 4).

## Discussion

The MMPs have proteolytic activity and break down the extracellular matrix, promoting angiogenesis, and controlling the growth and metastasis of tumor cells [21, 22]. They are also associated with the initiation, invasion, and metastasis of BC [4]. In the present study, the MMP-11 expression was shown to be significantly higher in BC cases compared to benign breast tumors. Several studies have observed MMP-11 expression at higher levels in BC than in nearby normal breast tissues [11, 15, 23–25]. MMP11 hindered SMAD family member 2 from being degraded in the tumor growth factor signaling pathway, which facilitated the growth of BC [25]. Low levels of CD8 + T cells, CD4 + T cells, and B cells are also correlated with high MMP-11 expression [26]. The MMPs also increase the availability of growth factors and cytokines [21] that could play a role in cancer initiation and progression.

In this study, there was a higher mRNA expression of MMP-2 and MMP-9 in BC patients compared to benign breast tumors, but no statistical significance. Other studies observed, higher levels of MMP-2 expression in BC than in nearby non-cancerous tissues [11, 23, 27, 28]. The significant link between increased angiostatin and the upregulation of MMP-2 and MMP-9 [29], suggests possible involvement in cancer initiation, progression, and invasion.

The current study found that the expression of MMP-11 in BC was about 2.4 times higher in lymph node-positive than in lymph node-negative. The MMP-11 increased cell motility of oral cancer cells through the focal adhesion kinase/SRC kinase pathway [30], and it is plausible that this pathway could be involved in BC metastasis. The expression of MMP-2 was about 1.6 times higher in BC patients with lymph nodes positive than in lymph nodes negative in this study. Increased cell migration and invasion are promoted by interactions between the tumor cell surface epidermal growth factor (EGF) receptors and its ligand EGF via upregulating MMP-2 expression [31].

The MMP-11 and MMP-9 mRNA expressions were higher in grade III tumors than in grade I-II in the current investigation. Similar to this study, grade III BC has been associated with increased MMP-11 mRNA expression [15]. The MMPs may promote tumor spread, invasion, and growth in BC by destroying cytokines and cell adhesion molecules and increasing angiogenesis and growth factors [12], which may lead to a worse prognosis.

The expression level of mRNA of MMP-11 was 5.7 times higher in ER-positive BC than in negative. Higher mRNA expression of MMP-11 in ER and PR-positive BC than negative BC is a finding supported by other studies [15, 25]. Cell survival mediated by MMP-11 depends on the p42/p44 MAPK and AKT pathway [32]. According to Marino et al. (2006), the primary transcriptional factor that interacts with ER and promotes the recruitment of coactivators is specificity protein 1 [33], specificity protein 1 is also implicated in the basal production of MMP-11 [34].

According to this study, HER2-negative BC had higher levels of MMP-2 and MMP-11 mRNA expression than HER2-positive BC. In contrast, other studies reported HER2-positive BC with increased mRNA expression of MMP-11 [25, 35]. The role of MMP-11 in HER2-positive BC through interaction with cancer cells, monocytes, and endothelial cells is also indicated [36].

The expression of MMP-9 and MMP-11 was higher in luminal A-like than in other BC subtypes. The higher immunohistochemical protein expression of MMP-9 among luminal A-like BC was also reported in another study [37]. In contrast, high levels of MMP-9 protein expression were found in triple-negative [14] and HER 2 enriched BC [18].

In general, our result showed MMP-11, which is a member of the stromelysin subgroup, has a stronger association with BC progression than MMP-2 and MMP-9. The MMP-11 is secreted in its active form [38], suggesting that MMP-11 may play a unique role in early tissue remodeling processes in BC progression. MMP-11 has also a significant role in tumor cell survival rather than in proteolytic action [22, 39], which may be another reason for the high expression of MMP-11 in BC progression. The BC stromal cells, particularly peritumoral fibroblasts, express significant levels of MMP-11 and are maybe associated with the early stages of aggressiveness of BC [40, 41].

## Conclusions

The present study showed an association between the mRNA expression MMPs and BC. In particular, MMP-11, but also MMP-2, and MMP-9 were higher in BC when compared with benign breast tumors. Of note, the MMP-11, MMP-2, and MMP-9 mRNA expression was significantly increased in lymph node-positive and estrogen receptor-positive BC. The MMP-11 and MMP-9 expressions were higher in grade III and luminal A-like tumors than in grade I-II and other subtypes, respectively. The HER2-negative BC had higher levels of MMP-2 and MMP-11 expression than HER2-positive BC. However, our findings suggest an important impact of MMPs in BC pathophysiology, particularly MMP-11, which therefore should be analyzed more in detail.

### Limtation of the study

The small sample size, retrospective design, and lack of study of additional MMP markers were the investigation’s key drawbacks.

### Electronic supplementary material

Below is the link to the electronic supplementary material.

Supplementary Material 1

## Data Availability

The data generated in this study are available within the article. Raw data were generated and processed from the authors and are available on request to the corresponding authors.
