# Supplementary material for: The expression of matrix metalloproteinase 2, 9 and 11 in Ethiopian breast cancer patients
Source: BMC Res Notes. 2023 Oct 5;16:253. doi: 10.1186/s13104-023-06518-5 (PMC10557335; doi:10.1186/s13104-023-06518-5)

**See Additional file 1**. Expression of MMP-2 in cases of benign and malignant breast cancer categorized by KI-67+ cell percentage, grade, and lymph node status. Log transformed values with median are denoted by horizontal lines.


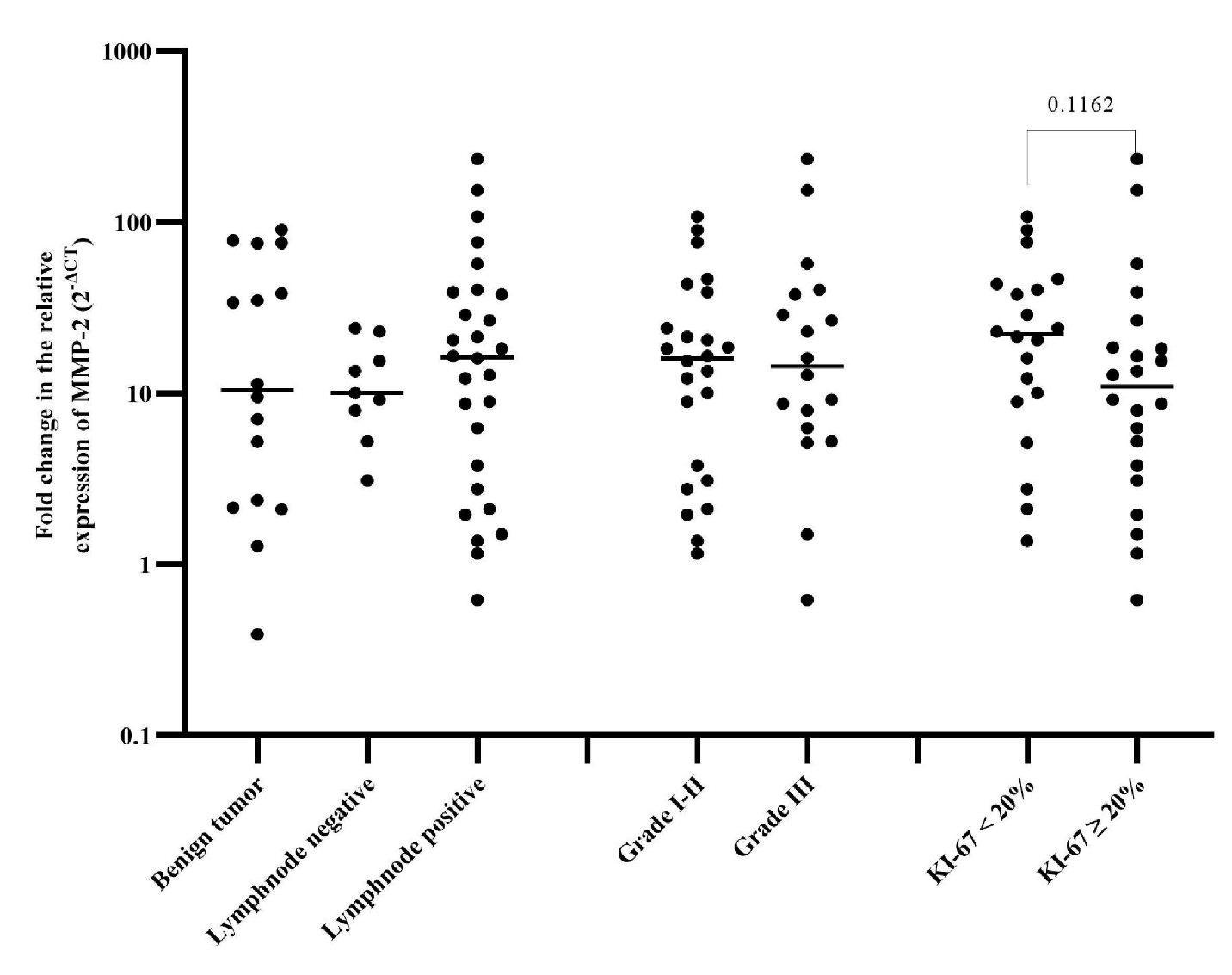


**See Additional file 2**. Expression of MMP-2 in cases of benign and malignant breast cancer categorized by ER, PR, HER-2 status, and IHC-defined breast cancer subtypes. Log transformed values with median are denoted by horizontal lines.


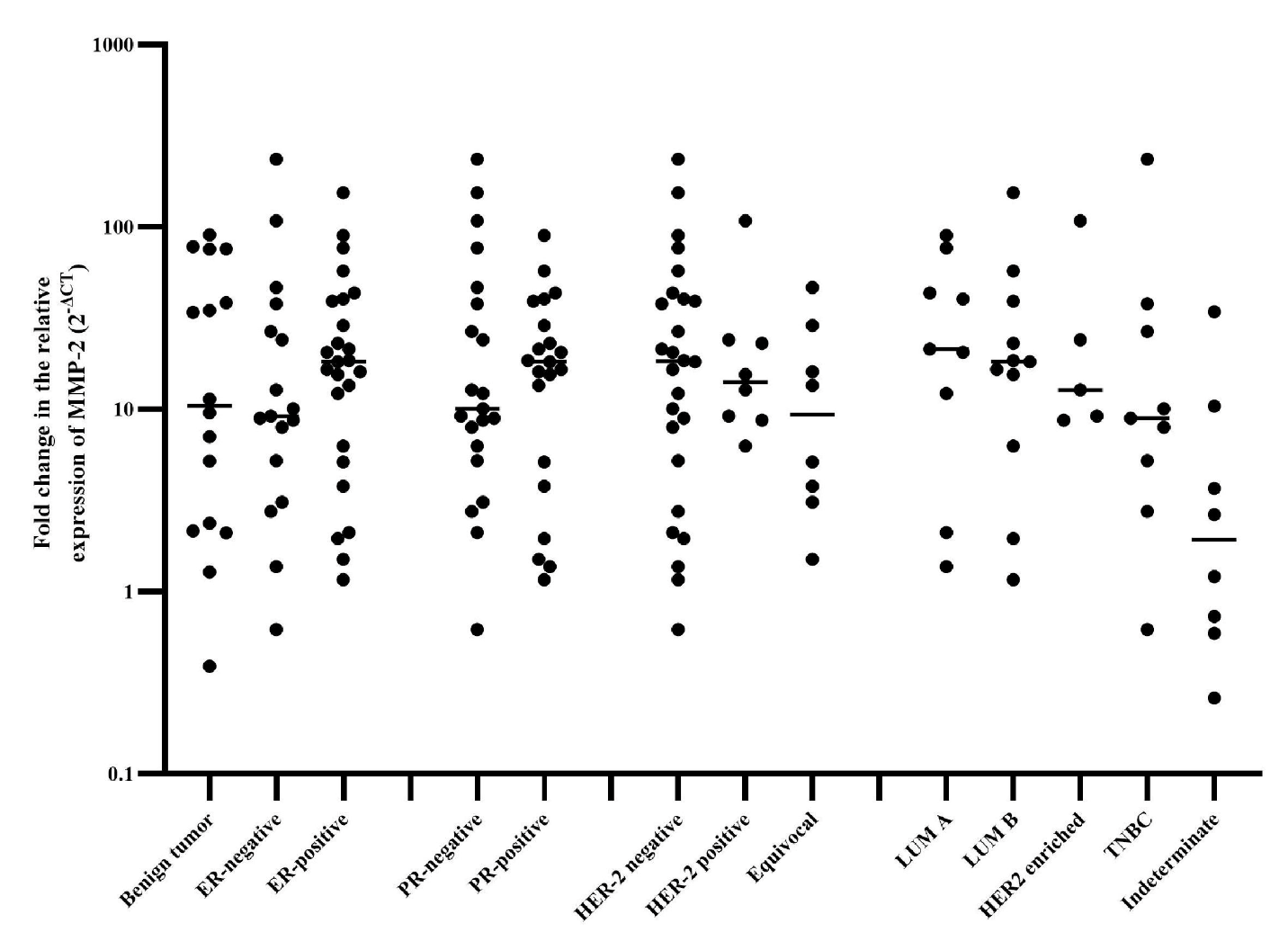


**See Additional file 3**. Expression of MMP-9 in cases of benign and malignant breast cancer categorized by KI-67+ cell percentage, grade, and lymph node status. Log transformed values with median are denoted by horizontal lines.


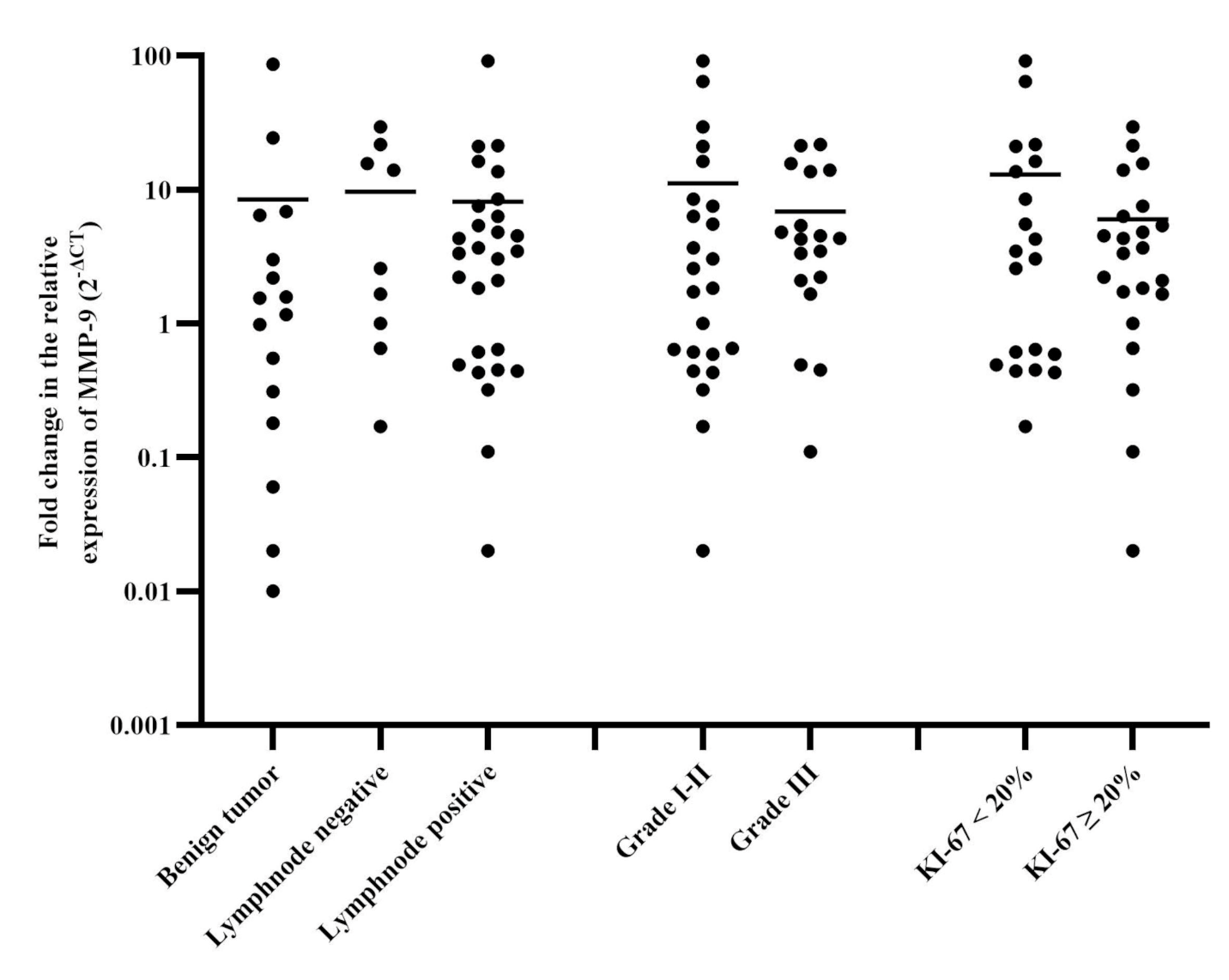


**See Additional file 4**. Expression of MMP-9 in cases of benign and malignant breast cancer categorized by ER, PR, HER-2 status, and IHC-defined breast cancer subtypes. Log transformed values with median are denoted by horizontal lines.


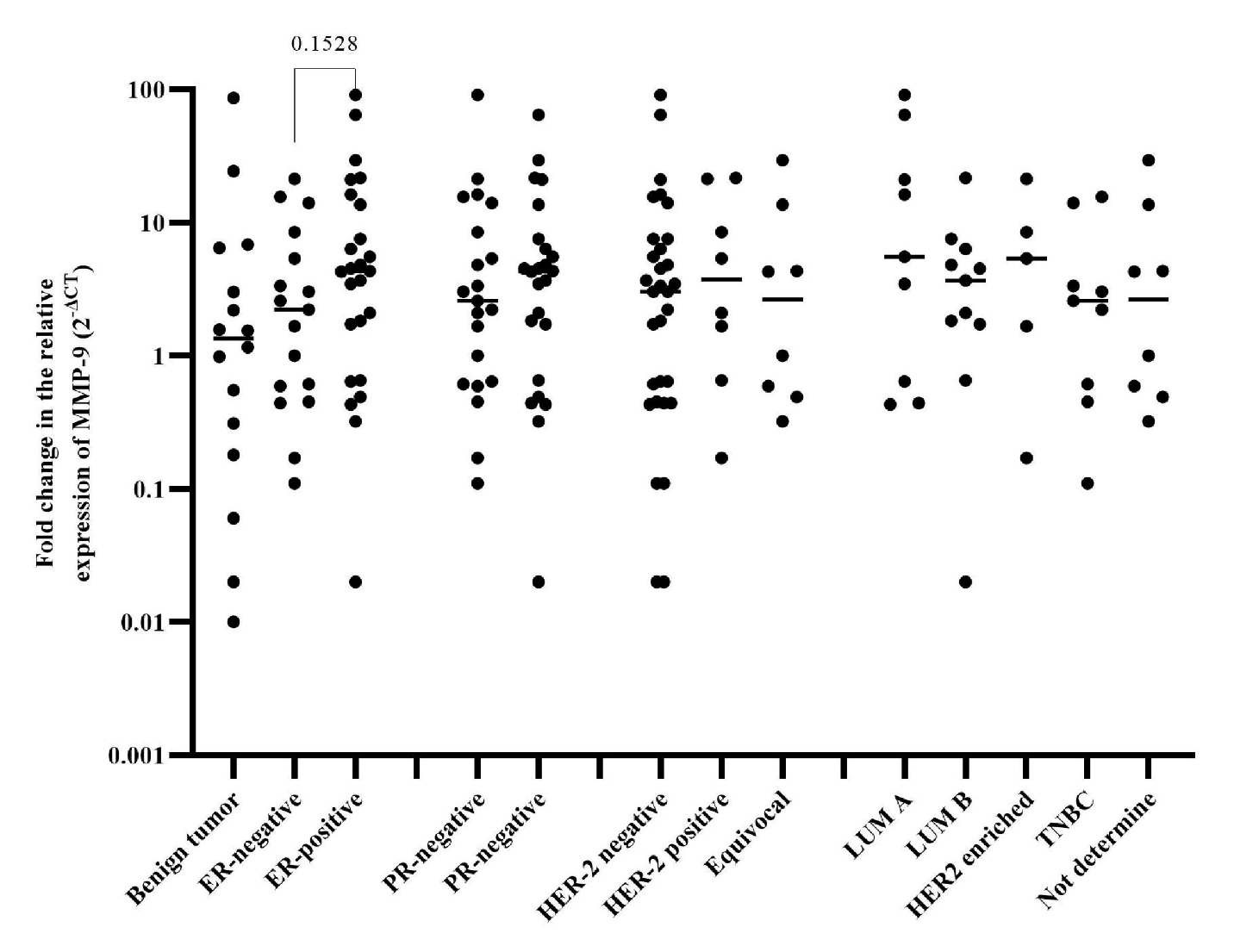

Supplement: Supplementary file 1 — Supplementary Material 1 [file 13104_2023_6518_MOESM1_ESM.docx]
